# Elevated Serum and Cerebrospinal Fluid CD138 in Patients With Anti-*N*-Methyl-d-Aspartate Receptor Encephalitis

**DOI:** 10.3389/fnmol.2019.00116

**Published:** 2019-05-16

**Authors:** Jiajia Zhu, Yongqi Li, Dong Zheng, Zhanhang Wang, Suyue Pan, Jia Yin, Honghao Wang

**Affiliations:** ^1^Department of Neurology, Nanfang Hospital, Southern Medical University, Guangzhou, China; ^2^Department of Otolaryngology Head and Neck Surgery, Third Affiliated Hospital of Sun Yat-sen University, Guangzhou, China; ^3^Department of Neurology, The Affiliated Brain Hospital of Guangzhou Medical University, Guangzhou, China; ^4^Department of Neurology, 999 Brain Hospital, Guangzhou, China

**Keywords:** anti-NMDAR encephalitis, CD138, cerebrospinal fluid, cytokine, modified Rankin Scale, syndecan-1

## Abstract

**Background:**

CD138 (also known as syndecan-1) is an important component of endothelial cell glycocalyx, and it is reportedly involved in negative regulation of various inflammatory processes. The clinical implications of circulating and cerebrospinal fluid (CSF) soluble CD138 (sCD138) in patients with Anti-*N*-methyl-d-aspartate receptor (NMDAR) encephalitis remain unclear.

**Objective:**

The aim of the current study was to investigate associations between serum and CSF sCD138 levels in anti-NMDAR encephalitis patients.

**Methods:**

The participants enrolled in the study included 27 with anti-NMDAR encephalitis, 11 with viral meningoencephalitis, and 22 controls. At acute stage and 3 to 6-month follow-up time-points, sCD138, tumor necrosis factor-α, matrix metalloproteinase-2, and matrix metalloproteinase-9 in serum and CSF were measured in all participants via enzyme-linked immunosorbent assays.

**Results:**

Serum and CSF levels of sCD138 were significantly increased in patients with anti-NMDAR encephalitis. Furthermore, after 3–6 months of follow-up CSF sCD138 levels were significantly decreased in anti-NMDAR encephalitis patients. Changes in sCD138 levels were significantly associated with amelioration of modified Rankin Scale scores in patients with anti-NMDAR encephalitis.

**Conclusion:**

In anti-NMDAR encephalitis patients, high circulating, and CSF sCD138 is associated with inflammation and poor clinical prognosis. The present study suggests that sCD138 may be an informative biomarker of inflammation in anti-NMDAR encephalitis.

## Introduction

Anti-*N*-methyl-d-aspartate receptor (anti-NMDAR) encephalitis, a newly characterized antibody-mediated disorder of the central nervous system formally recognized in 2007, has been increasingly identified as a cause of autoimmune and paraneoplastic or non-paraneoplastic encephalitis (Peery et al., 2012). The precise mechanisms of its pathogenesis are yet to be fully elucidated. Neuroinflammation and dysfunction of the blood brain barrier (BBB) may play critical roles. Activation of cytokines can lead to disruption of the BBB in NMDAR-rich regions (Peng et al., 2019). NMDAR can subsequently be exposed to active immune recognition, inducing anti-NMDAR encephalitis.

CD138 (also known as syndecan-1) is predominantly expressed on the surfaces of plasma cells, endothelial cells, and epithelial cells (O’Connell et al., 2004), and soluble CD138 is a marker of endothelial glycocalyx degradation (Rehm et al., 2007). High circulating soluble CD138 due to endothelial glycocalyx degradation is associated with inflammation, high sympathoadrenal activity, coagulopathy, and increased mortality in cases of multiple myeloma (Ren et al., 2018), diabetic nephropathy (Svennevig et al., 2006), acute graft-versus-host disease (Seidel et al., 2003), dialysis (Kowalewska et al., 2016), and major vascular surgery (Johansson et al., 2011). In a previous study, glycocalyx degradation led to BBB dysfunction and brain edema after asphyxia cardiac arrest in rats (Zhu et al., 2018). CD138 is also expressed in plasma cells (Voyvodic et al., 2014). Several studies suggest that CD138 may be involved in the pathology of experimental autoimmune encephalitis. Increased levels have been reported in cases of neuromyelitis optica (Pei et al., 2018).

Blood brain barrier permeability injury and activation of inflammation in the central nervous system is the main mechanism of pathogenesis of NMDAR encephalitis. Martinez-Hernandez et al. (2011) reported that abundant CD138^+^ cells (plasma cells/plasmablasts) were observed in perivascular regions and interstitial spaces (Martinez-Hernandez et al., 2011). Research conducted by DellaValle et al. (2018) suggested that glycocalyx degradation was a new biomarker for neurological autoimmune diseases (DellaValle et al., 2018). Thus, CD138 may have a modulatory role in the transendothelial migration of monocytes, and it may contribute to the formation of inflammatory lesions (Zhang et al., 2013). Given the critical role of the endothelium in BBB dysfunction and outcomes in cases of encephalitis, endothelial dysfunction in anti-NMDAR encephalitis has attracted much attention. To our knowledge, however, few studies have investigated the pathological effects of CD138 in anti-NMDAR encephalitis. The aims of the present study were to investigate CD138 levels in anti-NMDAR encephalitis patients and evaluate possible associations between CD138 and modified Rankin Scale (mRS) scores.

## Materials and Methods

### Study Population

Twenty-seven patients with anti-NMDAR encephalitis fulfilling the diagnostic criteria described in Graus et al. (2016) were included in the study. The diagnosis of anti-NMDAR encephalitis was confirmed based on clinical manifestations and the detection of anti-NMDAR antibodies in cerebrospinal fluid (CSF) via cell-based assays. Patients were excluded if any of the following conditions that can potentially influence serum CD138 levels were present: preexisting overt coronary artery disease (typical angina, myocardial infarction), transient ischemic attack, stroke, vasculitis, pregnancy, or active infection. For comparison, 22 patients with non-inflammatory neurologic disorders were selected as controls (10 with peripheral neuropathy, 12 with movement disorders) and 12 patients with viral meningoencephalitis (VM) who fulfilled the diagnostic criteria were also included. All patients were recruited from the Department of Neurology of Nanfang Hospital, Southern Medical University, in Guangzhou, China.

The demographic data derived from patients with anti-NMDAR encephalitis, viral meningitis, and controls are shown in Table 1. Clinical manifestations were categorized into the following groups for further analysis: prodromal symptoms (such as headache and fever), psychiatric symptoms, memory deficits, speech disturbances, seizures, movement disorders, impaired consciousness, sleep disorders, and central hypoventilation. All patients and controls underwent lumbar puncture for CSF analysis within 3 days of admission, and patients with anti-NMDAR encephalitis underwent a follow-up lumbar puncture for CSF reevaluation 3–6 months after discharge. All patients underwent tumor screening via computed tomography, magnetic resonance imaging, or B-scan ultrasonography at least once during their hospital stay. Each patient’s neurological status was assessed using the mRS score at the most critical time and at a 3 to 6-month follow-up time-point after discharge. Treatments included first-line immunotherapy, second-line immunotherapy, and tumor removal. First-line immunotherapies included the use of steroids, intravenous immunoglobulins, or plasma exchange alone or combined, and second-line immunotherapies included rituximab, azathioprine, or cyclophosphamide treatment alone or combined. Treatment outcome was recorded as “failed” if: (i) no sustained improvement occurred within 3 months of the initiation of immunotherapy or tumor removal; and/or if (ii) mRS score remained at four or higher.

**TABLE 1:** The clinic manifestations and baseline characteristics of anti-NMDAR encephalitis and controls.

**Characteristic**	**Anti-NMDAR encephalitis**	**Viral meningitis**	**Controls**
Gender (female/male)	15/12	5/6	10/12
Age (years, mean ± SD)	34.67 ± 19.35	33.81 ± 16.38	37.45 ± 15.25
Max mRS (mean ± SD)	4.15 ± 0.76	NA	NA
3-month mRS (mean ± SD)	2.85 ± 1.02	NA	NA
**CSF routine**			
CSF WBC (×10^6^, median, range)	5 (125–0)	8 (80–2)	0 (10–0)
CSF TP (g/L, mean ± SD)	0.48 ± 0.47	0.63 ± 0.66	0.34 ± 0.24
**Symptom onset (n, %)**			
Prodromal symptoms	14 (51.9%)	0	NA
Psychiatric symptoms****	23 (85.2%)	2	NA
Memory deficits****	14 (51.9%)	3	NA
Seizures****	21 (77.8%)	1	NA
Movement disorders****	6 (22.2%)	1	NA
Central hypoventilation	6 (22.2%)	0	NA
**Treatment (n, %)**			
Sole first line treatment	16 (59.3%)	NA	NA
Combined first and second-line treatment	11 (40.7%)	NA	NA
**Tumor comorbidity (n, %)**			
Ovarian teratoma	2 (7.4%)	0	0
**CSF NMDAR antibody positive**	33 (100%)	0	0

### Enzyme-Linked Immunosorbent Assay

Cerebrospinal fluid samples were centrifuged immediately after collection to isolate cells and larger particles, then stored at −80°C until they were used in enzyme-linked immunosorbent assays (ELISAs). Commercial sandwich ELISA kits were used to quantify CD138 (Quantikine ELISA, R&D Systems) and the inflammatory cytokines tumor necrosis factor (TNF)-α (Cusabio, Wuhan, China), matrix metalloproteinase (MMP)-2, and MMP-9 (Bender MedSystems GmbH Campus, Vienna, Austria) in accordance with the manufacturers’ instructions.

### Follow-Up Evaluation

A total of 15 patients underwent follow-up evaluation 3–6 months after discharge for the assessment of mRS scores. CSF levels of CD138, TNF-α, MMP-2, and MMP-9 were also determined.

### Statistical Analysis

All statistical analyses were performed using SPSS version 20.0 (IBM Corp., Armonk, NY, United States). Mann–Whitney *U*-tests were performed to assess differences in CD138, TNF-α, MMP-2, and MMP-9 levels between patients with anti-NMDAR encephalitis and controls. Spearman test was performed to evaluate correlations between CSF cytokines and mRS scores, and *p* < 0.05 was deemed to indicate statistical significance.

## Results

### Demographic and Clinical Features of Anti-NMDAR Encephalitis Patients

The demographic data derived from patients with anti-NMDAR encephalitis (*n* = 27), VM (*n* = 11), controls and healthy controls (*n* = 22) are shown in Table 1. Notably (i) diagnoses in all patients with confirmed anti-NMDAR encephalitis included detection of anti-NMDAR autoantibody in CSF; and (ii) mRS scores in patients with anti-NMDAR encephalitis at the most critical time dropped significantly after 3–6 months of follow-up, indicating a probable effect of treatment in patients with anti-NMDAR encephalitis.

### CD138 and Cytokine Levels Increased in Serum and CSF in Patients With Anti-NMDAR Encephalitis

**FIGURE 1:**
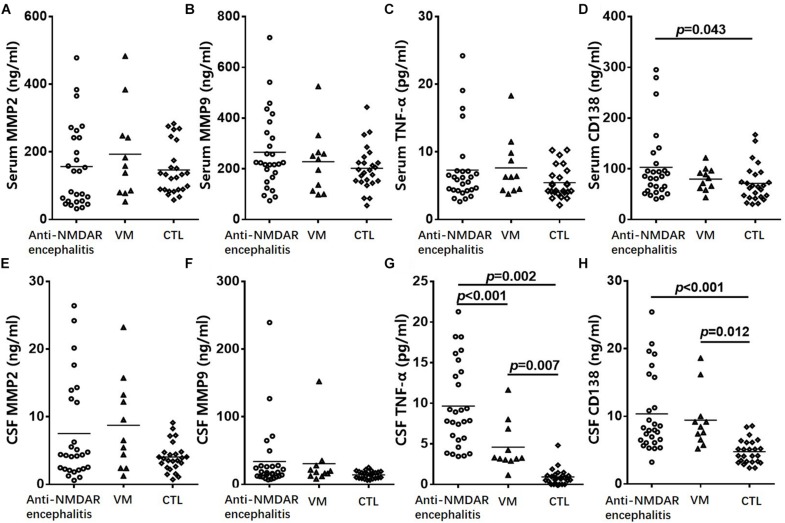
Change of CD138 and cytokine levels in serum and CSF in patients. Serum levels of MMP-2 **(A)**, MMP-9 **(B)**, and TNF-α **(C)** in patients with anti-NMDAR encephalitis did not differed significantly from the corresponding levels in VM patients or healthy controls. The levels of serum CD138 **(D)** in anti-NMDAR encephalitis patients were significantly higher than those of VM patients and healthy controls. CSF levels of MMP-2 **(E)** and MMP-9 **(F)** in patients with anti-NMDAR encephalitis had no significant difference from the corresponding levels in VM patients or healthy controls. CSF levels of TNF-α **(G)** and CD138 **(H)** in patients with anti-NMDAR encephalitis were significantly higher than those of VM patients and controls. The *p*-values were indicated within figures. MMP-2, matrix metalloproteinase-2; MMP-9, matrix metalloproteinase-9; TNF-α, tumor necrosis factor-α; VM, viral meningoencephalitis; anti-NMDAR, Anti-*N*-methyl-d-aspartate receptor; CSF, cerebrospinal fluid.

To investigate the role of CD138, serum and CSF levels of CD138 in patients with anti-NMDAR encephalitis (*n* = 27), VM (*n* = 13), and healthy controls (*n* = 20) were measured via ELISAs. As shown in Figure 1, the levels of both serum and CSF CD138 in anti-NMDAR encephalitis patients at peak periods of disease were significantly higher than those of VM patients (*p* = 0.043) and healthy controls (*p* < 0.001).

To further evaluate the role of humoral immunity and BBB permeability injury in anti-NMDAR encephalitis, we measured the serum and CSF levels of TNF-α, MMP-2, and MMP-9 via ELISAs. CSF levels of TFN-α in patients with anti-NMDAR encephalitis were significantly higher than those of VM patients (*p* = 0.002) and controls (*p* < 0.001). However, neither serum nor CSF levels of MMP-2 or MMP-9 in patients with anti-NMDAR encephalitis differed significantly from the corresponding levels in VM patients or healthy controls.

### Altered CSF Levels of Inflammatory Cytokines in Anti-NMDAR Encephalitis

**FIGURE 2:**
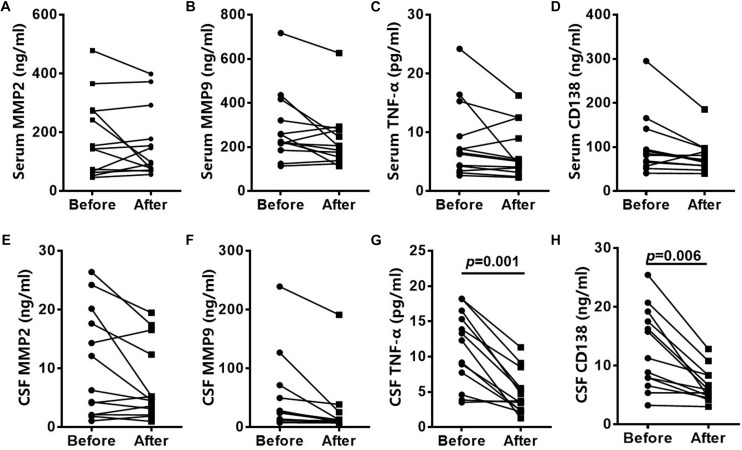
Altered CSF levels of inflammatory cytokines in patients with anti-NMDAR encephalitis. CD138, TNF-α, MMP2, and MMP9 levels in serum of anti-NMDAR encephalitis patients had no significantly difference in 3- to 6-month follow-up **(A–D)**. CSF levels of CD138 and TNF-α in anti-NMDAR encephalitis patients were significantly lower than peak values at the 3- to 6-month follow-up time-point. CSF levels of MMP-2 and MMP-9 did not exhibit significant reduction **(E–H)**. The *p*-values were indicated within figures. MMP-2, matrix metalloproteinase-2; MMP-9, matrix metalloproteinase-9; TNF-α, tumor necrosis factor-α; anti-NMDAR, Anti-*N*-methyl-d-aspartate receptor; CSF: cerebrospinal fluid.

At the 3 to 6-month follow-up time-point CSF levels of CD138 in patients were significantly lower than peak values (*p* = 0.006), as were TNF-α levels (*p* = 0.001) (Figure 2). CSF levels of MMP-2 and MMP-9 did not exhibit significant reduction at the 3 to 6-month follow-up time-point.

### Serum CD138, CSF CD138, and Clinical Parameters in Anti-NMDAR Encephalitis

**FIGURE 3:**
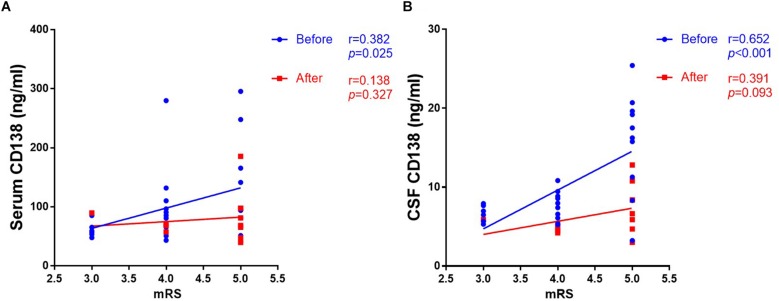
Correlation between levels of CD138 in serum and CSF and mRS scores in patients with anti-NMDAR encephalitis. The levels of both serum **(A)** and CSF **(B)** CD138 in anti-NMDAR encephalitis patients at peak periods of disease were significantly correlated with mRS scores in 3- to 6-month follow-up. There was not a correlation between CD138 levels in serum and CSF at follow up and mRS scores. The *p*-values were indicated within figures. mRS, modified Rankin Scale; anti-NMDAR, Anti-*N*-methyl-d-aspartate receptor; CSF, cerebrospinal fluid.

As shown in Figure 3, the levels of both serum (*r* = 0.382, *p* = 0.025) and CSF (*r* = 0.652, *p* < 0.001) CD138 in anti-NMDAR encephalitis patients at peak periods of disease were significantly correlated with mRS scores in 3 to 6-month follow-up. There was not a correlation between CD138 levels in serum and CSF at follow up and mRS scores.

## Discussion

In the present study serum and CSF CD138 levels were elevated in anti-NMDAR encephalitis patients and reflected disease activity. These data suggest that serum and CSF CD138 may be useful as a biomarker of active brain inflammation in anti-NMDAR encephalitis.

Some recent studies have shown that BBB dysfunction is involved in the main pathophysiological mechanisms of anti-NMDAR encephalitis (Ehrenreich, 2017). It has previously been reported that inflammatory factors are increased in the CSF of patients with anti-NMDAR encephalitis (Chen et al., 2018; Ding et al., 2018). Heparan sulfate proteoglycans such as CD138 play multiple roles during inflammation and BBB injury. Zhang et al. (2013) have reported increased leukocyte recruitment to the brain in CD138 knockout mice (Zhang et al., 2013). Pro-inflammatory phenotype conversion in loss of CD138 endothelial cells response to inflammation has also been reported (Holzmann et al., 2018). Elevated levels of soluble CD138 have been detected in the blood of patients with sepsis (Smart et al., 2018), post-cardiopulmonary resuscitation syndrome (Grundmann et al., 2012), multiple myeloma (Seidel et al., 2000), and systemic lupus erythematosus (Kim et al., 2015), and patients undergoing dialysis (Vlahu et al., 2012) and major vascular surgery (Rehm et al., 2007).

CD138 has two distinct effects in patients with anti-NMDAR encephalitis. The increase in serum CD138 may be due to degradation of endothelial glycocalyx. BBB permeability reportedly increased after glycocalyx degradation (Johansson et al., 2014). There is initial recruitment of B cells, and inflammatory mediators enter the brain via the impaired BBB, which is an important component of the mechanism of activation of brain inflammation. Moreover, increased CD138 levels, as a marker of CD138^+^ cells in CSF, suggest the activation of inflammation in the brain (Martinez-Hernandez et al., 2011).

Transmembranous CD138 is a proteoglycan located on the luminal surface of endothelial cells, and forms the backbone of the endothelial glycocalyx (Reitsma et al., 2007) which is involved in the regulation of vascular adhesiveness and permeability (Sieve et al., 2018). Circulating CD138 is considered a marker of endothelial glycocalyx degradation. Elevated serum CD138 in patients with anti-NMDAR encephalitis may reflect damage to the brain endothelial glycocalyx, which constitutes the BBB (Kutuzov et al., 2018). Inflammatory factors such as TNF-α can reportedly induce severe degradation of the glycocalyx (Ando et al., 2018). The results of the present study suggest that the glycocalyx is degraded during the pathogenesis of anti-NMDAR encephalitis, and that this may be an early pathophysiological process. Increased levels of inflammatory factors such as interleukin 6 in the blood suggest the activation of inflammation in peripheral circulation in patients with anti-NMDAR encephalitis (Byun et al., 2016). CD138 is also a highly specific marker of plasma cells and plasmablasts (Akl et al., 2015). Elevated levels of CSF CD138 indicate CD138^+^ plasma cell entry into the parenchyma, and the activation of neuroinflammation in the brain. Martinez-Hernandez et al. (2011) also reported that CD138^+^ plasma cells were often detected in perivascular, interstitial, and Virchow-Robin spaces in NMDAR-associated encephalitis lesions (Martinez-Hernandez et al., 2011; Macher et al., 2018). We speculate that damage to the endothelial glycocalyx after inflammation may lead to activated B cells crossing the BBB via leaky regions.

Collectively, our studies suggest that CD138 has a key role in endothelial activation and the regulation of inflammation in the brain. Initial systemic immune activation mediates glycocalyx degradation, and inflammatory factors and activated immune cells enter the brain via an impaired BBB. These results need to be confirmed in a longitudinal study involving a large cohort of patients with anti-NMDAR encephalitis.

## Ethics Statement

The study protocol was approved by the Ethics Committee of Nanfang Hospital, Southern Medical University, each participant provided written informed consent to participate.

## Author Contributions

HW, YJ, and SP conceived this study and designed the experiments. JZ, DZ, and ZW collected the samples and clinical data. JZ and YL performed the experiments, analyzed the data, and wrote the manuscript. YL had re-performed all the data analyses and revised the manuscript. All authors read and approved the final version of the manuscript and agreed to submit it for publication.

## Conflict of Interest Statement

The authors declare that the research was conducted in the absence of any commercial or financial relationships that could be construed as a potential conflict of interest.
